# An audit comparing telephone reviews to standard face-to-face consultations within child and adolescent mental health services at Massereene House

**DOI:** 10.1192/bjo.2021.281

**Published:** 2021-06-18

**Authors:** Eileen Moss

**Affiliations:** Massereene House, NHSCT, St Georges University of London

## Abstract

**Aims:**

This audit was carried out in response to the Coronavirus pandemic. The COVID-19 pandemic has forced many teams to review how they provide care to their patients. Due to attempting to reduce the spead of COVID-19, the Child and Adolescent Mental Health Service within the Northern Health and Social Care Trust largely switched to telephone reviews instead of face-to-face reviews for non-urgent outpatient appointments from March 2020 onwards. The aim of this audit was to establish whether or not service users found telephone reviews to be as useful and therapeutic as the previous standard face-to-face reviews.

**Method:**

A questionnaire was used to assess opinions on telephone reviews. Those who were answering the questions were asked to rate their answers on the following scale: “not at all”, “a little”, “somewhat” or “a great deal”. There was an “any other comments” section at the end where service users could give detailed opinions on how successful they thought telephone reviews were. A sample of twenty patients was involved. This cohort of twenty patients was a mixture of ten ADHD reviews and ten medication reviews. The audit was conducted by one person and this was done via the telephone.

**Result:**

For questions one to four (which will be fully outlined in the poster), the most popular category chosen was “somewhat” and this indicates that the majority of patients found telephone reviews somewhat better than face-to-face appointments. For question five (which was- “Overall, was the help you received good?”), 80% of service users stated that the help that they received was “a great deal” better than the help that they had received at previous face-to-face appointments. Lastly, for question six (which was- “If a friend or family member needed similar help, would you recommend that they are phoned by our service?”), 80% of service users said that they would recommend our service “a great deal” to family members or friends.

**Conclusion:**

Generally the feedback was positive for the telephone reviews. However, some still outlined a preference for face-to-face reviews. There may have been bias in this audit as it was the same doctor who did the telephone reviews as conducted the audit. To conclude, telemedicine is likely to become more popular in the future especially as the Coronavirus pandemic is still currently a worldwide problem therefore it is important to explore how service users feel about this as a way of communicating with the clinicians who are treating them.

